# Changes in Metabolic Syndrome Severity Following Individualized Versus Standardized Exercise Prescription: A Feasibility Study

**DOI:** 10.3390/ijerph15112594

**Published:** 2018-11-20

**Authors:** Ryan M. Weatherwax, Joyce S. Ramos, Nigel K. Harris, Andrew E. Kilding, Lance C. Dalleck

**Affiliations:** 1 Auckland University of Technology, Human Potential Centre, Auckland 0632, New Zealand; nigel.harris@aut.ac.nz; 2 Western Colorado University, Recreation and Exercise & Sport Science, Gunnison, CO 81231, USA; ldalleck@western.edu or lance.dalleck@flinders.edu.au; 3 Colorado Mountain College, Health and Wellness, Aspen, CO 81611, USA; 4 SHAPE Research Centre, Exercise Science and Clinical Exercise Physiology, College of Nursing and Health Sciences, Flinders University, Adelaide 5000, Australia; joyce.ramos@flinders.edu.au; 5 Centre for Research on Exercise, Physical Activity and Health, School of Human Movement and Nutrition Sciences, The University of Queensland, Brisbane 4072, Australia; 6 Sports Performance Research Institute New Zealand, Auckland University of Technology, Auckland 0632, New Zealand; andrew.kilding@aut.ac.nz

**Keywords:** training responsiveness, MetS z-score, personalized medicine, exercise non-responders, ventilatory threshold, HRR

## Abstract

This study sought to investigate the efficacy of standardized versus individualized exercise intensity prescription on metabolic syndrome (MetS) severity following a 12-week exercise intervention. A total of 38 experimental participants (47.8 ± 12.2 yr, 170.7 ± 8.0 cm, 82.6 ± 18.7 kg, 26.9 ± 6.7 mL·k^−1^·min^−1^) were randomized to one of two exercise interventions (exercise intensity prescribed using heart rate reserve or ventilatory threshold). Following the 12-week intervention, MetS z-score was significantly improved for the standardized (−2.0 ± 3.1 to −2.8 ± 2.8 [*p* = 0.01]) and individualized (−3.3 ± 2.3 to −3.9 ± 2.2 [*p* = 0.04]) groups. When separating participants based on prevalence of MetS at baseline and MetS *z*-score responsiveness, there were six and three participants in the standardized and individualized groups, respectively, with three or more MetS risk factors. Of the six participants in the standardized group, 83% (5/6) of the participants were considered responders, whereas 100% (3/3) of the individualized participants were responders. Furthermore, only 17% (1/6) of the participants with MetS at baseline in the standardized group no longer had symptoms of MetS following the intervention. In the individualized group, 67% (2/3) of participants with baseline MetS were not considered to have MetS at week 12. These findings suggest that an individualized approach to the exercise intensity prescription may ameliorate the severity of MetS.

## 1. Introduction

Metabolic syndrome (MetS) is the simultaneous occurrence of three or more cardiovascular disease risk factors including central obesity, hyperglycemia, hypertriglyceridemia, low high-density lipoprotein cholesterol (HDL-C), and hypertension, which elevates the risk of cardiovascular events [1]. Therefore, reducing the severity of this syndrome may serve as a target to improve global health. Indeed, an exercise-induced increase in cardiorespiratory fitness has been well established as a protective factor against individual risk factors constituting MetS [2], with high fit MetS individuals reported to have lower risk of cardiovascular mortality relative to less fit counterparts [3]. However, the results of previous studies have shown considerable individual variability in responses to a specific dose of standardized exercise, including the so-termed ‘responders’, ‘non-responders’, and, in some cases, ‘adverse responders’ [4,5]. This variability in response to exercise has been purported among others [4,6], to be attributed to the lack of a personalized approach to the exercise prescription [7].

It is believed that a more individualized approach to exercise prescription may better optimize training efficacy and thus limit training unresponsiveness [7]. Specifically, it has been reported that when exercise intensity is titrated according to a threshold-based model (i.e., ventilatory threshold), 100% of individuals showed favorable change in maximal oxygen uptake (VO_2_max) compared to only 41.7% when the exercise intensity was ‘standardized’ or prescribed according to a relative percent method (i.e., % heart rate reserve [HRR]) [8]. It has been suggested that the variability in response to a standardized exercise prescription may be due to the incapacity of this method to account for individual metabolic differences [9]. Furthermore, it should be noted that investigations thus far have only concentrated on the impact of different ‘short-term’ standardized exercise doses on the interindividual VO_2_max [4] and individual cardiometabolic responses [5]. Indeed, the specific interindividual ‘MetS severity’ changes in response to ‘individualized training’ have yet to be explored.

Moreover, in conjunction with the difficulty in titrating available intervention dosages to optimally treat or manage MetS, there is also a dilemma in determining the change in MetS severity to better account for the clinical significance of a particular intervention. The established categorical criteria of MetS proposed by different organizations (i.e., International Diabetes Federation [IDF]) [1] are often criticized due their incapacity to account for improvement in a certain MetS component if the magnitude of change is not large enough to deviate from a qualifying category. For example, a systolic blood pressure reduction from 142 mmHg to 131 mmHg following an intervention would still classify as a MetS risk factor according to the IDF criteria (systolic blood pressure (SBP) ≥130 mmHg), regardless of achieving a clinically significant reduction [10]. For this reason, a continuous risk score assessment known as the MetS z-score was introduced to better acknowledge MetS risk factor changes and thus MetS severity, following clinical interventions [11]. Interestingly, similar to previous findings [12], Earnest et al. [11] reported highly fit individuals to have lower MetS severity, which was depicted as a reduced MetS z-score relative to less fit counterparts.

The aim of this study was to investigate the efficacy of standardized versus individualized exercise prescription on MetS severity over a 12-week program. We hypothesized that individualized exercise intensity prescription would reduce the severity of MetS more than the standardized intervention in a greater proportion of individuals.

## 2. Materials and Methods

A total of 49 sedentary men and women who were interested in starting an exercise intervention and improving their health and well-being were recruited as experimental participants from a community wellness program and the surrounding community through advertisement at the local university, newspaper, and word-of-mouth. In order to be considered for the investigation, the participants needed the following inclusion criteria: low to moderate risk based on the American College of Sports Medicine Standards [13], participation in less than 30 minutes of moderate intensity physical activity three days a week or less, and between the ages of 30–75 years. Participants were excluded from the investigation if they showed signs or symptoms suggestive of pulmonary, cardiovascular, or metabolic conditions determined from a standard medical history questionnaire and intake interview. Similar to previous research [14,15], a convenience sample (20 control participants) separate from the experimental participants was used to take into consideration the moral and ethical considerations of withholding a known physiological and psychological benefit of an exercise intervention. Therefore, control participants were recruited by finding individuals who were interested in the various health measurements from the baseline and 12-week laboratory testing and met the same inclusionary criteria, but were not interested in increasing physical activity. Control participants were encouraged to maintain their current dietary intake and physical activity habits following the baseline laboratory testing.

Written informed consent was obtained from all of the participants prior to initiation of the study. The current investigation is a secondary analysis of a larger randomized control trial. A description of the study methodology and rationale has been previously published in detail [16]. The Auckland University of Technology Ethics Committee (16/264) and the Western State Colorado University Institutional Review Board (HRC2016-01-90R6) have approved the methodology for this study.

### 2.1. Experimental Testing

At baseline and week 12, all of the primary outcome variables were obtained. In order to mitigate possible changes due to the time of day at which testing took place, baseline and post-program testing occurred on the same day of the week and time of day, to the best of our ability. Participants were instructed to refrain from consumption of food or drink, other than water, and partaking in strenuous exertion for the 12 h leading up to the laboratory testing. All of the post-program testing took place within approximately two to three days of the last exercise training session.

### 2.2. Analysis of Dietary and Physical Activity Habits

Participants verbally agreed to not change their regular nutritional intake habits, and at baseline and post-program, they completed a three-day dietary recall (two weekdays and one weekend day). Either prior to the baseline testing when initial contact and interest in the study was provided or at the first baseline testing appointment, participants were provided a nutritional log with columns identifying day of the week/time eaten, a prompt to provide as much detail about the food or beverage as possible (type, brand, restaurant eaten at, etc.), the amount eaten, how the food was prepared, and whether they added fat, salt, or sugar to the food or drink. Participants were encouraged to complete the dietary recall throughout the day as they ate and drank food to increase the accuracy of the information received. If they were actively logging food with all of the desired information, the participant was allowed time during the initial baseline assessment to record the last two weekdays and the closest weekend day to the food log. In order to establish energy intake, percentages of macronutrients, and grams of macronutrients, participants recorded as much detail about the food and drink consumed on each of the three days.

Also at baseline and post-intervention, participants completed the International Physical Activity Questionnaire (IPAQ) to identify physical activity levels (metabolic equivalents [MET] min·wk^−1^) and sedentary behavior per day (i.e., time spent sitting). The researching completing the baseline testing went through each of the prompts to explain what was being asked and help the participants identify the time and associated intensity level of the activity. In some instances, further discussion was needed with participants to identify whether sedentary behavior inclusionary criteria at baseline were met for experimental participants at baseline and post-program for control participants.

### 2.3. Anthropometric and Resting Measurements

Participants were weighed to the nearest 0.1 kg on a medical grade scale and measured height to the nearest 0.5 cm using a stadiometer (Tanita Corporation WB-3000, Tokyo, Japan). Waist circumference was measured at the narrowest horizontal circumference above the umbilicus and below the xiphoid process to the nearest 0.5 cm [13]. Resting heart rate (HR) followed standardized procedures [13]. Participants sat with back support for five minutes with their feet on the ground and arms supported near heart level. A medical-grade pulse oximeter (Nonin Medical Inc., Plymouth, MN, USA) was used to establish resting HR following the five minutes of rest. Following assessment of resting HR and while still in the same seated position, blood pressure was assessed using a standard stethoscope and sphygmomanometer (American Diagnostic Corporation Diagnostic 700 Series, Hauppauge, NY, USA) to determine left arm blood pressure (BP). The average of two consecutive resting measurements separated by one minute were used.

### 2.4. Blood Profile Measurements

The Cholestech LDX system (Alere Inc., Waltham, MA, USA), which has been shown to have excellent reproducibility [17,18], was used to analyze all of the fasting lipid and blood glucose measurements. All of the analyses were performed based on manufacturer guidelines. In summary, an optics check was completed prior to all of the blood analyses. Participants washed their hands thoroughly with soap, rinsed with warm water, and the skin was subsequently wiped with an alcohol swab and allowed to dry. On the distal end of the third digit of the right hand, a lancet was used to puncture the finger, and the free-flowing blood sample was collected using a 40-μL capillary tube without milking the finger. The blood sample was then transferred from the capillary tube into the commercially available test cassette for analysis. Measurements of total cholesterol, high-density lipoprotein cholesterol (HDL), triglycerides (TG), and blood glucose (BG) were obtained. Blood sample were disposed of based on standard biohazard procedures.

### 2.5. Maximal Exercise Test and Verification Protocol

A graded exercise test (GXT) using a modified Balke pseudo-ramp protocol on a motorized treadmill (Powerjog GX200, Maine, USA) was completed to determine maximal oxygen consumption (VO_2_max) and threshold measurements using a participant chosen self-selected pace. Following a four-minute warm-up with the workload gradually increasing to the starting self-selected speed and an incline of 0%, the incline was then increased by 1% each min until volitional fatigue was reached. During the GXT, HR was monitored using a chest strap and radiotelemetric device (Polar Electro, Woodbury, NT, USA) and expired air and gas exchange data using a metabolic analyzer (Parvo Medics TrueOne 2.0, Salt Lake City, UT, USA) were continuously recorded and monitored. Before the GXT, the metabolic analyzer was calibrated with a calibration gas mixture (16.00% O_2_ and 4.00% CO_2_) and room air (20.93% O_2_ and 0.03% CO_2_) in accordance with manufacturer guidelines and the instructional manual. Following the GXT, the last 15 s of gas analysis data were averaged and considered to be the final data point. Subsequently, the 15 s of gas exchange data occurring before the final data point were also averaged. The mean of the two processed data points represented the VO_2_max for the GXT. The highest HR reached during the GXT was considered the maximal HR, and HRR was calculated by taking the difference between the maximal and resting HR.

A supramaximal verification protocol was used to confirm that a ‘true’ VO_2_max was achieved using methods previously published [19,20]. In summary, 20 minutes following the completion of the GXT, participants were asked to complete a four-minute warm-up followed by a volitional test to fatigue at a constant workload that was 5% higher than the last completed stage of the GXT. During the verification protocol, HR and gas exchange data were monitored, and VO_2_max was determined based on the same protocols as the GXT. A ‘true’ VO_2_max was confirmed if the two calculated VO_2_max values from the GXT and verification protocol were within ±3.0%, which was the measurement error of the metabolic analyzer. If a participant had a difference in VO_2_max values >3.0%, they were asked to repeat the GXT and verification bout protocol within 24–72 h until a difference less than ±3.0% was achieved to confirm ‘true’ VO_2_max reached.

The determination of the first (VT1) and the second ventilatory threshold (VT2) were performed based on previously published methods [8,16,21]. In summary, a visual inspection of the gas exchange data from the GXT were analyzed to determine VT1 and VT2 using time, ventilatory equivalents of O_2_ (VE/VO_2_), and ventilatory equivalents of CO_2_ (VE/VCO_2_). VT1 occurred when VE/VO_2_ increased without a concurrent increase in VE/VCO_2_, whereas VT2 was the point that both VE/VO_2_ and VE/VCO_2_ simultaneously increased.

### 2.6. Exercise Prescription and Training

Prior to baseline testing, participants were randomized into one of the two experimental groups according to a computer-generated sequence of random numbers stratified by sex. Participants exercised three days a week for 12 weeks following detailed descriptions published elsewhere [16]. Exercise intensity (i.e., heart rate) was established based on percentages of heart rate reserve based on the heart rates at VT1 and VT2 occurring during the GXT. Exercise volume was equated for each group based on energy expenditure per kg of body weight per week (kcal·kg^−1^·wk^−1^) to ensure an isocaloric volume among groups. The prescribed HR was correlated with a VO_2_ from the GXT to establish a low and high exercise volume and converted to a minimum range per exercise session.

During the exercise intervention, participants rested in a seated position for five minutes when they arrived at the laboratory. Subsequently, resting heart rate and blood pressure was recorded follow methods outlined before to ensure that drastic changes in these measures were not noted. Following the resting measurements, participants warmed up at a self-selected pace with self-selected increases in the exercise workload for five minutes, at which point the prescribed exercise intensity was reached. Participants then exercised for the prescribed duration (i.e., time) based on the calculated energy expenditure derived from values obtained during the GXT with continuous monitoring of the heart rate using a chest strap and radiotelemetric receiver (Polar Electro, Woodbury, NY, USA). Furthermore, exercise heart rate was checked by a research assistant at approximately one-third and two-thirds through the total time to ensure that the intensity prescription was adhered. At the end of the exercise session, participants completed a five-minute cool-down with decreasing workloads until the heart rate was within 15 bpm of resting values.

### 2.7. Establishment of Metabolic Syndrome z-Score

A continuous risk score assessment scale (MetS z-score) has been previously used to identify changes in MetS risk factors following an exercise intervention [22]. The MetS severity was presented as a sex-specific MetS z-score, which was previously validated to evaluate cardiometabolic risk in middle-aged men and women [23]. The MetS z-score was calculated using the following equations, [24] where FG = fasting glucose; HDL = high-density lipoprotein cholesterol; MAP = mean arterial pressure; TG = triglycerides; and WC = waist circumference:

Men=[(40−HDL)÷8.9]+[(TG−150÷69)+[(FG−100)÷17.8]+[(WC−102)÷11.5]+[(MAP−100)÷10.1]

Women=[(50−HDL)÷14.5]+[(TG−150÷69)+[(FG−100)÷17.8]+[(WC−88)÷12.5]+[(MAP−100)÷10.1]

### 2.8. Establishment of Metabolic Syndrome Responsiveness Criteria

A coefficient of variability (i.e., biological variability) for the MetS *z*-score was developed as a sub-group from the current investigation. At baseline, 15 participants completed the two baseline testing sessions, as described previously, no sooner than 24 h and no more than seven days later, while maintaining their current lifestyle. The MetS *z*-score was determined for both testing sessions and were subsequently used to determine a coefficient of variability (CV) based on previously published protocols [22]. In order for a participant to be considered a responder to improvements in MetS variables, they would need to have a MetS z-score change greater than the established MetS *z*-score CV in a favorable direction.

### 2.9. Statistical Analysis

All of the statistical analyses were performed using SPSS Version 25.0 (Chicago, IL, USA). Data were reported as mean ± standard deviation (SD). One-way analysis of variance (ANOVA) testing was used to compare groups at baseline and the Tukey post hoc test was used when appropriate. The assumption of normality was confirmed by an examination of normal plots of the residuals in ANOVA models and Shapiro–Wilk tests [25]. A two-way analysis of covariance (two-way ANCOVA) was used to analyze the between-group difference of the change in main dependent variables (i.e., SBP, diastolic blood pressure (DBP), MAP, HDL, TG, fasting BG, and WC) from baseline to 12 weeks, with the week-12 values as the dependent variables, and the baseline value as a covariate. A subsequent post hoc analysis with a comparison of the main effects and a Bonferroni adjustment was completed when appropriate. Analysis of within-group differences in continuous variables was completed using paired sample *t*-tests.

Delta values (Δ) for MetS z-scores were expressed as the week-12 value minus the baseline value to establish the change (Δ) in Mets z-score. The calculated laboratory-specific biological variability value (0.6) was compared to the Δ in MetS to determine MetS responsiveness. Subsequently, participants were categorized as either a ‘1’ = responder (Δ > 0.6) or ‘0’ = non-responder (Δ ≤ 0.6). Chi-square (χ^2^) tests were used to analyze the incidence of responders and non-responders for MetS *z*-score following the intervention separated by experimental group (standardized and individualized) and a Cramer’s V test to determine effect size.

## 3. Results

A total of 38 experimental participants who completed all of the testing sessions and had an exercise adherence of 82.9% ± 5.9% and 86.1% ± 4.7% for the standardized and individualized groups, respectively, were analyzed. Eleven experimental participants were not included in the final data analyses due to unrelated medical issues (*n* = 3), falling below the 70% adherence (*n* = 4), self-withdrawal (*n* = 3), and insufficient blood profile data (*n* = 1). There was considerable attrition for the control group, with only eight of the 20 recruited participants completing all of the exercise testing sessions due to increased physical activity and exercise habits following the baseline testing session. The reasons for not including the 12 participants in the control group in the final data analyses were due to unrelated medical issues (*n* = 2) and self-withdrawal (*n* = 10). 

Physical and physiological characteristics at baseline and post-program (week 12) are presented in Table 1. At baseline, there was a significant difference in SBP (mmHg) [F(2,43) = 3.85, *p* = 0.29] and VO_2_max (mL·k^−1^·min^−1^) [F(2,44) = 3.64, *p* = 0.035]. Post hoc analysis indicated that the mean SBP for the individualized group (119.7 ± 7.2 mmHg) was significantly lower than that of the standardized group (126.7 ± 9.9 mmHg), and the VO_2_max for the standardized group (24.4 ± 4.7 mL·kg^−1^·min^−1^) was significantly lower than that of the individualized group (29.5 ± 7.5 mL·kg^−1^·min^−1^). However, there were no other significant between-group differences at baseline across the experimental and control groups for all of the other variables. Adherence to the prescribed exercise intensity and duration for both experimental groups was excellent. In only one instance (week 3 for exercise duration in the standardized group) did the actual exercise that was performed differ from what was prescribed for that week.

### 3.1. Changes in MetS z-Score and MetS Variables

Following the 12-week intervention, the MetS z-score was significantly improved for both experimental groups with improvements of −2.0 ± 3.1 to −2.8 ± 2.8 [t(18) = 3.01, *p* = 0.01] and −3.3 ± 2.3 to −3.9 ± 2.2 [t(18) = 2.28, *p* = 0.04] for the standardized and individualized groups, respectively. When analyzing the individual components of the MetS z-score for the standardized group, there were significant reductions in the SBP of 126.7 ± 9.9 to 120.6 ± 6.5 mmHg [t(18) = 4.06, *p* = 0.001], DBP [t(18) = 2.31, *p* = 0.03] of 80.3 ± 11.1 to 74.7 ± 7.9 mmHg, MAP [t(18) = 3.07, *p* = 0.01] of 95.8 ± 9.9 to 90.0 ± 6.6 mmHg, and waist circumference [t(18) = 6.95, *p* = 1.7 × 10^−6^] 94.1 ± 1 5.8 to 90.9 ± 15.1 cm. For the individualized group, there were significant reductions in SBP [t(18) = 2.18, *p* = 0.04] of 119.7 ± 7.2 to 115.6 ± 8.9 mmHg and waist circumference [t(18) = 5.63, *p* = 2.4 × 10^−5^] of 88.0 ± 12.1 and 84.5 ± 11.0 cm. When investigating the mean scores for components of the MetS *z*-score at baseline in the standardized group, it was found that fasting BG, HDL, and TG each were in a healthy category. In the individualized group, the mean scores for the following components of the MetS *z*-score were within a healthy category at baseline: DBP, MAP, TG, fasting BG, and HDL.

### 3.2. Incidence of MetS z-Score Responders and Non-Responders

**Figure 1:**
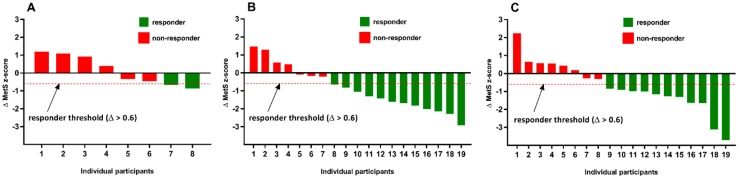
Metabolic syndrome (MetS) *z*-score responsiveness (Δ) for the control (**A**), standardized (**B**), and individualized (**C**) groups following 12 weeks of exercise training. The dashed line indicates the threshold of minimum change (Δ > 0.6) in a favorable direction that was required to be considered a responder.

The incidence of MetS z-score responders and non-responders in both the standardized and individualized groups are shown in Figure 1. In the standardized group, 63% (12/19) of participants were considered responders with a favorable change in MetS z-score (Δ > 0.6), and 37% (7/19) were considered non-responders with an undesirable change in MetS z-score. Similarly, in the individualized group, 58% (11/19) of participants were responders, and 42% (8/19) were considered non-responders for changes in MetS z-score. Based on the χ^2^ analysis, there was not a significant difference in the incidence of response (*p* = 0.74; Cramer’s V = 0.54) of exercise training strategy on MetS z-score responsiveness.

When separating participants based on the prevalence of MetS at baseline and MetS *z*-score responsiveness, there were six and three participants in the standardized and individualized groups, respectively, who were considered to have MetS. Of the six participants in the standardized group, 83% (5/6) of the participants were considered responders, whereas 100% (3/3) of participants in the individualized group were considered to be responders. Furthermore, only 17% (1/6) of the participants with MetS at baseline in the standardized group no longer had symptoms of MetS following the intervention. In the individualized group, 67% (2/3) of participants with baseline MetS were not considered to have MetS at week 12. In the control group, only 13% (1/8) of participant at baseline had three or more factors associated with MetS. However, at 12 weeks, 28% (3/8) of participants had MetS. The specific changes that occurred on an individual level in MetS *z*-score components for non-responders in both experimental groups are presented in Table 2. This table highlights how participants can be labeled as non-responders, with unfavorable changes occurring in the components of the MetS *z*-score, even though that measure remained in a healthy category.

### 3.3. Changes to Other Parameters

Changes in body mass index (BMI), weight, resting HR, and maximal HR were not significantly different within or between either experimental group following the 12-week exercise intervention. Furthermore, there was not a significant difference in overall dietary intake nor for each macronutrient at baseline and 12 weeks in any of the groups. There was a significant increase in physical activity from 836 ± 975 to 3680 ± 1671 MET·min^−1^·wk^−1^ [t(18) = −5.68, *p* = 2.2 × 10^−5^] and 937 ± 587 to 3855 ± 2261 MET·min^−1^·wk^−1^ [t(18) = −5.28, *p* = 5.1 × 10^−5^] for the standardized and individualized groups, respectively. However, this increase in activity was expected, since the final prescribed exercise duration was 1847 ± 442 MET·min^−1^·wk^−1^ and 2647 ± 892 MET·min^−1^·wk^−1^ for the standardized and individualized groups, respectively. Furthermore, the time spent sitting significantly decreased from 5.6 ± 2.7 to 4.5 ± 2.3 h·d^−1^ [t(18) = 2.11, *p* = 0.05] and 6.3 ± 2.4 to 5.4 ± 2.4 h·d^−1^ [t(18) = 2.40, *p* = 0.03].

The verification procedure following the GXT confirmed VO_2_max at baseline and post-program for all participants (46/46). The individual differences between the GXT and verification procedure at baseline and 12 weeks is presented in Table 1. Both experimental groups had significant improvements in absolute and relative VO_2_max in comparison to the control group. However, there was not a significant difference between the two experimental groups. Indeed, within-group differences showed that relative VO_2_max significantly improved from 24.4 ± 4.7 to 26.2 ± 4.2 mL·kg^−1^·min^−1^ [t(18) = −4.46, *p* = 3.1 × 10^−4^] and 29.2 ± 7.5 to 32.8 ± 8.6 mL ·kg^-1^·min^-1^ [t(18) = −9.86, *p* = 1.1 × 10^−8^] for the standardized and individualized groups, respectively. Absolute VO_2_max in the standardized group significantly improved from 2.0 ± 0.6 to 2.2 ± 0.6 L·min^−1^ [t(18) = −4.70, *p* = 1.8 × 10^−4^], and significant improvements in the individualized group were seen with a change of 2.4 ± 0.8 to 2.6 ± 0.9 L·min^−1^ [t(18) = −6.45, *p* = 1.0 × 10^−6^]. There was a greater overall percent change for the individualized compared to the standardized group (11.4 ± 3.7% compared to 7.7 ± 8.3%).

## 4. Discussion

To our knowledge, this is the first study to investigate the impact of two isocaloric exercise interventions with exercise intensity prescribed either on a standardized (%HRR) or individualized basis (threshold-based model using ventilatory threshold) on the severity of MetS. As expected, both exercise interventions resulted in more responders to a positive change in MetS severity (Δ > 0.6), which was depicted as a change in the MetS *z*-score, compared to control (Figure 1A). However, in contrast with our hypothesis, our results showed a similar percentage of individuals who significantly reduced MetS severity following both exercise interventions (standardized, 63% [12/19] versus individualized, 58% [11/19]). Interestingly, albeit based on a limited sample size, for those participants diagnosed with MetS at baseline, there was a greater number of individuals who reversed the syndrome following the individualized exercise intervention (66, 2/3) compared to the standardized (17%, 1/6) exercise prescription.

Previously, Johnson et al. [26] found that exercise volume plays a critical role in ameliorating MetS severity when exercise is performed at a low amount (~19 km·wk^−1^) with moderate intensity (40–55% VO_2_peak) for ~175 min·wk^−1^ or a high amount (~32 km·wk^−1^) with high intensity (65–80% VO_2_peak) for ~175 min·wk^−1^ compared to a low amount (~19 km·wk^−1^) with high intensity (65–80% VO_2_peak) for ~115 min·wk^-1^ over an eight-month period. In the current investigation, our data support the previous volume and MetS severity findings, with the standardized group increasing exercise volume to 168 ± 27 min·wk^−1^. However, our data is also inconsistent with these previous findings, with the individualized group increasing exercise volume to only 131 ± 32 min·wk^−1^. Indeed, Ramos et al. [22] reported a reduction in MetS severity following two methods of high-intensity interval training (HIIT) and continuous moderate intensity training. They found that a low-volume HIIT (51 min·wk^−1^) was as effective as a high-volume HIIT (114 min·wk^−1^) and moderate intensity continuous exercise (150 min·wk^−1^) following a 16-week intervention. One of the proposed mechanisms lessening the severity of MetS in the low-volume HIIT group was a due to a similar improvement in cardiorespiratory fitness (i.e., VO_2_max). Therefore, with both experimental groups significantly improving VO_2_max, even though they were not significantly different between groups, we believe that this is one potential mechanism underpinning why no difference in MetS severity reduction was seen between groups. Furthermore, it has been shown that individuals with greater insulin resistance, which individuals with diagnosed MetS are likely to have relative to those without the syndrome, are more sensitive to any dose of exercise [11]. This is supported by our findings, which showed no difference in the proportion of MetS individuals who responded to either an individualized or standardized exercise dose. Indeed, we found that of the six participants in the standardized group, 83% (5/6) of the participants were considered responders, whereas 100% (3/3) of the individualized participants were responders. Thus, the difference in the number of individuals diagnosed with MetS at baseline in the standardized group (*n* = 6) versus the individualized group (*n* = 3) may have caused our inability to detect a significant difference in MetS *z*-score change between groups.

It was not surprising that further improvements in some of the MetS *z*-score criteria were not seen due to the health status of each group at baseline. For example, the standardized group was in a healthy range for fasting BG, HDL, and TG, and the individualized group was in a healthy range for DBP, MAP, TG, fasting BG, and HDL. Therefore, both groups had a limited ability to improve MetS criteria, especially in the individualized group, due to a ‘ceiling effect’ [7]. Moreover, as exhibited in Table 2, a non-response in various individual components of the MetS *z*-score may not result in an unfavorable change to cardiometabolic health. Indeed, Dalleck et al. [27] found that an adverse cardiometabolic response (i.e., a response in an unfavorable direction by two times the technical error) rarely resulted in an increased 10-year cardiovascular disease risk. These findings are important to recognize, because even if a participant has an unfavorable change in a MetS risk factor, it may not always be associated with an increased risk overall, especially if other cardiometabolic and cardiovascular factors are improving.

An individualized exercise prescription has been shown to increase the training responsiveness of VO_2_max following an exercise intensity prescription based on ventilatory thresholds compared to a standardized approach using HRR [8,21]. Indeed, unpublished data from the current investigation supports these previous findings with 60% and 100% of standardized and individualized participants, respectively, being considered VO_2_max responders to a site-specific and cohort-specific technical error. Therefore, an individualized program appears to be superior to a standardized program when evaluating changes in VO_2_max due to the driving force being the exercise intervention itself. Given the reported cardioprotective benefits conferred by higher levels of fitness (i.e., improved VO_2_max) [3], these findings have important public health implications. However, when investigating changes in cardiometabolic risk factors (i.e., changes in MetS factors), it has been established that the exercise intervention as well as the lifestyle outside of the exercise intervention play a critical role in the changes in MetS factors. For example, dietary intake [28,29], sedentary behavior [30], sitting for prolonged periods [31], sleep duration [32], and chronic stress [33] have all been linked to influencing components of MetS syndrome. Moreover, heightened psychological stress and inadequate sleep have been linked to a variety of negative health factors, including altered endocrine function, increased sympathetic and decreased parasympathetic tone, increased blood pressure, and increased insulin and glucose levels [34,35,36]. Furthermore, psychological stress and insufficient sleep have been linked to a reduction in post-exercise recovery [37,38]. In the present study, the individualized group, while not statistically significant, had an overall increase in caloric intake from baseline to post-intervention, and sedentary behavior (time spent sitting) remained about an hour more than the standardized group. Unfortunately, we did not delineate the overall time spent sedentary compared to continuous durations of time engaged in sedentary behavior (i.e., five hours of sedentary behavior in one setting or 30 minutes of sedentary behavior 10 times throughout the day). Indeed, these two factors and those previously mentioned but not accounted for in the investigation may have been influential factors in the overall MetS *z*-score responsiveness at the individual level.

We believe the present study highlights the feasibility of using a MetS *z*-score to quantify cardiometabolic training responsiveness. However, future research should investigate a standardized and individualized approach to exercise intensity prescription in individuals with known MetS at baseline, control for sedentary behavior and frequency of interrupting sedentary behavior throughout the intervention, have a standardized dietary intake for participants leading up to blood analysis, and include a more comprehensive training responsiveness criterion (i.e., incorporation of MetS z-score and VO_2_max responsiveness criteria).

### Limitations

With this being a feasibility study, there are several limitations that should be noted. There was an overall lack of participants with known MetS in both groups; however, many participants in both groups had one or more cardiometabolic risk factors at baseline. Therefore, the present results cannot be generalized to clinical populations at this point. Further research is warranted to identify differences in the standardized and individualized exercise intensity prescription methodologies in a MetS-specific cohort. The participant pool represented a standard ‘exercise clinic demographic’, but there may be heterogeneity in the results due to the large age range used. There were many lifestyle and psychological factors that were not accounted for and could have had an impact on the overall findings. Furthermore, men were underrepresented, only accounting for 24% of the participants. Additionally, while dietary intake was analyzed at baseline and post-intervention, there was not a standardized dietary protocol prior to blood analysis, which may have accounted for some of the changes in individual blood profiles. Moreover, survey data were used in this study to analyze dietary intake/habits and physical activity levels at baseline and post-program. This methodology itself may have limited the overall interpretations of the findings. Both of these methods relied on the honesty of the participants to accurately record nutritional data and quantify their activity levels. These surveys were only conducted on two occasions. Incorporating more frequent analyses of these measures would have allowed for a better understanding of whether changes in nutrition and activity levels (outside of the prescribed exercise intervention) occurred. Lastly, hydration status during testing, exercise, and changes from pre-intervention to post-intervention were not assessed.

## 5. Conclusions

Our findings suggest that MetS *z*-score significantly improved following 12 weeks of standardized and individualized exercise intensity prescription. While there was no significant difference in individual MetS *z*-score responsiveness, it appears that an individualized approach may ameliorate the severity of MetS in individuals with three or more cardiometabolic risk factors at baseline. Further research is warranted on whether an individualized exercise prescription approach further reduces MetS severity when compared to a standardized approach in individuals with known MetS.

## Figures and Tables

**Table 1:** Physical and physiological characteristics and dietary intake at baseline and 12 weeks for standardized, individualized, and control groups.

Parameter	Control (*n* = 8; Women = 6, Men = 2)		Standardized (*n* = 19; Women = 15, Men = 4)		Individualized (*n* = 19; Women = 14, Men = 5)	
	Baseline	Week 12	Baseline	Week 12	Baseline	Week 12
Age (yr)	45.6 ± 7.9	-	51.2 ± 12.5	-	44.9 ± 11.4	-
Height (cm)	171.7 ± 6.4	-	169.2 ± 8.8	-	172.1 ± 7.1	-
Weight (kg)	75.3 ± 15.1	75.1 ± 14.6	84.5 ± 21.1	84.4 ± 20.7	80.6 ± 16.2	79.9 ± 15.2
BMI	25.5 ± 4.5	25.5 ± 4.6	29.3 ± 5.7	29.3 ± 5.4	27.1 ± 4.2	26.8 ± 3.8
WC (cm)	86.8 ± 10.8	86.2 ± 10.6	94.1 ± 15.8	90.9 ± 15.1 *	88.0 ± 12.1	84.5 ± 11.0 *^,†^
Systolic BP (mmHg)	119.8 ± 7.8	121.5 ± 11.0	126.7 ± 9.9	120.6 ± 6.5 *	119.7 ± 7.2 ^‡^	115.6 ± 8.9 *
Diastolic BP (mmHg)	82.0 ± 7.0	81.0 ± 10.8	80.3 ± 11.1	74.7 ± 7.9 *	76.1 ± 8.4	77.4 ± 6.8
MAP (mmHg)	94.6 ± 6.2	94.5 ± 9.8	95.8 ± 9.9	90.0 ± 6.6 *	90.6 ± 7.7	90.1 ± 6.8
TG (mmol·L^−1^)	1.9 ± 1.4	1.9 ± 1.6	1.4 ± 0.5	1.4 ± 0.6	1.3 ± 0.6	1.2 ± 0.6
Fasting BG (mmol·L^−1^)	5.1 ± 0.5	5.4 ± 0.5	5.2 ± 0.6	5.1 ± 0.7	5.1 ± 0.4	5.0 ± 0.5
HDL (mmol·L^−1^)	1.3 ± 0.4	1.4 ± 0.3	1.6 ± 0.6	1.6 ± 0.6	1.5 ± 0.5	1.6 ± 0.5
MetS *z*-Score	−1.4 ± 3.8	−1.3 ± 4.0	−2.0 ± 3.1	−2.8 ± 2.8 *	-3.3 ± 2.3	−3.9 ± 2.2 *
Caloric intake (kcal)	1327 ± 418	1265 ± 317	1520 ± 563	1518 ± 500	1539 ± 493	1555 ± 403
Carbohydrate (g)	136.5 ± 55.0	121.1 ± 41.8	160.4 ± 60.5	158.8 ± 63.9	168.2 ± 68.6	164.5 ± 57.2
Lipid (g)	56.0 ± 18.1	54.0 ± 11.9	61.1 ± 31.2	62.8 ± 26.4	68.6 ± 23.4	67.5 ± 13.6
Protein (g)	71.7 ± 43.6	55.0 ± 7.6	64.1 ± 16.4	63.8 ± 22.0	73.6 ± 36.6	64.8 ± 25.2
Carbohydrate (%)	40.6 ± 5.8	37.9 ± 5.6	41.7 ± 6.9	40.9 ± 7.8	43.1 ± 8.2	41.9 ± 6.7
Lipid (%)	38.7 ±7.5	39.2 ± 7.0	35.9 ± 9.2	37.1 ± 8.4	40.7 ± 7.9	40.6 ± 8.1
Protein (%)	22.1 ± 13.4	17.9 ± 3.1	18.2 ± 6.3	17.8 ± 5.1	19.6 ± 10.6	16.5 ± 3.8
PA (MET·min^−1^·wk^−1^)	1354 ± 1018	1176 ± 1109	838 ± 979	3680 ± 1671 *^,†^	937 ± 587	3855 ± 2261 *^,†^
Time Sitting (hours·d^−1^)	6.5 ± 1.2	6.9 ± 2.5	5.6 ± 2.7	4.5 ± 2.3 *^,†^	6.3 ± 2.4	5.4 ± 2.4 *
Resting HR (b·min^−1^)	74.1 ± 7.8	69.5 ± 7.5	70.4 ± 8.9	68.8 ± 7.7	68.8 ± 9.7	68.1 ± 11.4
Maximal HR (b·min^−1^)	173.9 ± 12.4	170.1 ± 11.1 *	166.4 ± 15.7	167.6 ± 15.5	170.1 ± 18.4	169.2 ± 14.4
VO_2_max (ml·kg^−1^·min^−1^)	28.4 ± 4.5	27.7 ± 4.6	24.4 ± 4.7	26.2 ± 4.2 *^,†^	29.5 ± 7.5 ^‡^	32.8 ± 8.6 *^,†^
VO_2_max (L·min^−1^)	2.2 ± 0.7	2.1 ± 0.7	2.0 ± 0.6	2.2 ± 0.6 *^,†^	2.4 ± 0.8	2.6 ± 0.9 *^,†^
% Diff in VO_2_max (GXT and Verification)	0.6 ± 1.5	0.0 ± 2.1	−0.2 ± 1.8	-0.4 ± 1.8	0.2 ± 1.7	-0.7 ± 1.7
% Δ in VO_2_max	-	−2.3 ± 8.5	-	7.7 ± 8.3 ^†^	-	11.4 ± 3.7 ^†^

Values are mean ± SD. BG, blood glucose; BMI, body mass index; BP, blood pressure; GXT, graded exercise test; HDL, high density lipoprotein; HR, heart rate; MAP, mean arterial pressure; MET, metabolic equivalents; PA, physical activity; TG, triglycerides; WC, waist circumference; Δ, change. * *p* ≤ 0.05 pre- to post-change within-group difference; ^†^ Significantly difference from control group; ^‡^ Significantly different from standardized group.

**Table 2:** A breakdown of all the individual changes in MetS *z*-score components for non-responders in both the standardized and individualized experimental groups.

Change in MetS z-Score Component	Number of Participants	
	Standardized	Individualized
SBP increased, but remained healthy	1	4
DBP increased, but remained healthy	1	4
TG increased, but remained healthy	5	4
BG increased, but remained healthy	2	3
HDL decreased, but remained healthy	3	4
WC increased, but remained healthy	1	0
DBP increased to an unhealthy range	1	2
TG increased to an unhealthy range	1	1
BG increased to an unhealthy range	1	1
HDL started and decreased in an unhealthy range	1	0

BG, fasting blood glucose; DBP, diastolic blood pressure; HDL, high-density lipoprotein; SBP, systolic blood pressure; TG, triglycerides; WC, waist circumference.

## References

[1] Alberti K.G., Eckel R.H., Grundy S.M., Zimmet P.Z., Cleeman J.I., Donato K.A., Fruchart J.-C., James W.P.T., Loria C.M., Smith S.C. (2009). Harmonizing the Metabolic Syndrome: A Joint Interim Statement of the International Diabetes Federation Task Force on Epidemiology and Prevention; National Heart, Lung, and Blood Institute; American Heart Association; World Heart Federation; International Atherosclerosis Society; and International Association for the Study of Obesity. Circulation.

[2] Warburton D.E.R., Nicol C.W., Bredin S.S.D. (2006). Health benefits of physical activity: The evidence. Can. Med. Assoc. J..

[3] Katzmarzyk P.T., Church T.S., Blair S.N. (2004). Cardiorespiratory Fitness Attenuates the Effects of the Metabolic Syndrome on All-Cause and Cardiovascular Disease Mortality in Men. Arch. Intern. Med..

[4] Williamson P.J., Atkinson G., Batterham A.M. (2017). Inter-Individual Responses of Maximal Oxygen Uptake to Exercise Training: A Critical Review. Sports Med..

[5] Bouchard C., Blair S.N., Church T.S., Earnest C.P., Hagberg J.M., Häkkinen K., Jenkins N.T., Karavirta L., Kraus W.E., Leon A.S. (2012). Adverse Metabolic Response to Regular Exercise: Is It a Rare or Common Occurrence?. PLoS ONE.

[6] Hecksteden A., Kraushaar J., Scharhag-Rosenberger F., Theisen D., Senn S., Meyer T. (2015). Individual response to exercise training—A statistical perspective. J. Appl. Physiol..

[7] Mann T.N., Lamberts R.P., Lambert M.I. (2014). High Responders and Low Responders: Factors Associated with Individual Variation in Response to Standardized Training. Sports Med..

[8] Wolpern A.E., Burgos D.J., Janot J.M., Dalleck L.C. (2015). Is a threshold-based model a superior method to the relative percent concept for establishing individual exercise intensity? A randomized controlled trial. BMC Sports Sci. Med. Rehab..

[9] Katch V., Weltman A., Sady S., Freedson P. (1978). Validity of the relative percent concept for equating training intensity. Eur. J. Appl. Physiol. Occup. Physiol..

[10] Staessen J.A., Wang J.-G., Thijs L. (2001). Cardiovascular protection and blood pressure reduction: A meta-analysis. Lancet.

[11] Earnest C.P., Artero E.G., Sui X., Lee D., Church T.S., Blair S.N. (2013). Maximal Estimated Cardiorespiratory Fitness, Cardiometabolic Risk Factors, and Metabolic Syndrome in the Aerobics Center Longitudinal Study. Mayo Clin. Proc..

[12] Blair S.N., Kampert J.B., Kohl H.W., Barlow C.E., Macera C.A., Paffenbarger R.S., Gibbons L.W. (1996). Influences of Cardiorespiratory Fitness and Other Precursors on Cardiovascular Disease and All-Cause Mortality in Men and Women. JAMA.

[13] American College of Sports Medicine (2014). ACSM’s Guidelines for Exercise Testing and Prescription.

[14] Astorino T.A., Edmunds R.M., Clark A., King L., Gallant R.A., Namm S., Fischer A., Wood K.M. (2017). High-Intensity Interval Training Increases Cardiac Output and VO2max. Med. Sci. Sports Exerc..

[15] Astorino T.A., Schubert M.M., Palumbo E., Stirling D., McMillan D.W., Cooper C., Godinez J., Martinez D., Gallant R. (2013). Magnitude and time course of changes in maximal oxygen uptake in response to distinct regimens of chronic interval training in sedentary women. Eur. J. Appl. Physiol..

[16] Weatherwax R.M., Harris N.K., Kilding A.E., Dalleck L.C. (2016). The incidence of training responsiveness to cardiorespiratory fitness and cardiometabolic measurements following individualized and standardized exercise prescription: Study protocol for a randomized controlled trial. Trials.

[17] Shephard M.D., Mazzachi B.C., Shephard A.K. (2007). Comparative Performance of Two Point-of-Care Analysers for Lipid Testing. Clin. Lab..

[18] Dale R.A., Jensen L.H., Krantz M.J. (2008). Comparison of Two Point-of-Care Lipid Analyzers for Use in Global Cardiovascular Risk Assessments. Ann. Pharmacother..

[19] Weatherwax R., Richardson T., Beltz N., Nolan P., Dalleck L. (2016). Verification Testing to Confirm VO2max in Altitude-Residing, Endurance-Trained Runners. Int. J. Sports Med..

[20] Dalleck L.C., Astorino T.A., Erickson R.M., McCarthy C.M., Beadell A.A., Botten B.H. (2012). Suitability of verification testing to confirm attainment of VO2max in middle-aged and older adults. Res. Sports Med..

[21] Dalleck L.C., Haney D.E., Buchanan C.A., Weatherwax R.M. (2016). Does a personalised exercise prescription enhance training efficacy and limit training unresponsiveness? A randomised controlled trial. J. Fit. Res..

[22] Ramos J.S., Dalleck L.C., Borrani F., Beetham K.S., Wallen M.P., Mallard A.R., Clark B., Gomersall S., Keating S.E., Fassett R.G. (2017). Low-Volume High-Intensity Interval Training Is Sufficient to Ameliorate the Severity of Metabolic Syndrome. Metab. Syndr. Relat. Disord..

[23] Viitasalo A., Lakka T.A., Laaksonen D.E., Savonen K., Lakka H.-M., Hassinen M., Komulainen P., Tompuri T., Kurl S., Laukkanen J.A. (2014). Validation of metabolic syndrome score by confirmatory factor analysis in children and adults and prediction of cardiometabolic outcomes in adults. Diabetologia.

[24] Malin S.K., Nightingale J., Choi S.-E., Chipkin S.R., Braun B. (2013). Metformin modifies the exercise training effects on risk factors for cardiovascular disease in impaired glucose tolerant adults: Metformin Modifies Exercise Training Effects. Obesity.

[25] Cohen J. (1988). Statistical Power Analysis for the Behavioral Sciences.

[26] Johnson J.L., Slentz C.A., Houmard J.A., Samsa G.P., Duscha B.D., Aiken L.B., McCartney J.S., Tanner C.J., Kraus W.E. (2007). Exercise Training Amount and Intensity Effects on Metabolic Syndrome (from Studies of a Targeted Risk Reduction Intervention through Defined Exercise). Am. J. Cardiol..

[27] Dalleck L., Van Guilder G., Richardson T., Vella C. (2015). The prevalence of adverse cardiometabolic responses to exercise training with evidence-based practice is low. Diabetes Metab. Syndr. Obes..

[28] McKeown N.M., Meigs J.B., Liu S., Saltzman E., Wilson P.W.F., Jacques P.F. (2004). Carbohydrate Nutrition, Insulin Resistance, and the Prevalence of the Metabolic Syndrome in the Framingham Offspring Cohort. Diabetes Care.

[29] Tortosa A., Bes-Rastrollo M., Sanchez-Villegas A., Basterra-Gortari F.J., Nunez-Cordoba J.M., Martinez-Gonzalez M.A. (2007). Mediterranean Diet Inversely Associated With the Incidence of Metabolic Syndrome: The SUN prospective cohort. Diabetes Care.

[30] Greer A.E., Sui X., Maslow A.L., Greer B.K., Blair S.N. (2015). The Effects of Sedentary Behavior on Metabolic Syndrome Independent of Physical Activity and Cardiorespiratory Fitness. J. Phys. Act. Health.

[31] Owen N., Healy G.N., Matthews C.E., Dunstan D.W. (2010). Too Much Sitting: The Population Health Science of Sedentary Behavior. Exerc. Sport Sci. Rev..

[32] Xi B., He D., Zhang M., Xue J., Zhou D. (2014). Short sleep duration predicts risk of metabolic syndrome: A systematic review and meta-analysis. Sleep Med. Rev..

[33] Kaur J. (2014). A Comprehensive Review on Metabolic Syndrome. Cardiol. Res. Pract..

[34] McEwen B.S. (2006). Sleep deprivation as a neurobiologic and physiologic stressor: Allostasis and allostatic load. Metabolism.

[35] Spiegel K., Leproult R., Van Cauter E. (1999). Impact of sleep debt on metabolic and endocrine function. Lancet.

[36] Dattilo M., Antunes H.K.M., Medeiros A., Mônico Neto M., Souza H.S., Tufik S., de Mello M.T. (2011). Sleep and muscle recovery: Endocrinological and molecular basis for a new and promising hypothesis. Med. Hypotheses.

[37] Stults-Kolehmainen M.A., Bartholomew J.B. (2011). Psychological stress impairs short-term muscular recovery from resistance exercise. Med. Sci. Sports Exerc..

[38] Samuels C. (2008). Sleep, Recovery, and Performance: The New Frontier in High-Performance Athletics. Neurol. Clin..

